# The Expansion of Animal MicroRNA Families Revisited

**DOI:** 10.3390/life5010905

**Published:** 2015-03-13

**Authors:** Jana Hertel, Peter F. Stadler

**Affiliations:** 1 Bioinformatics Group, Department of Computer Science, and Interdisciplinary Center for Bioinformatics, University Leipzig, Härtelstrasse 16–18, D-04107 Leipzig, Germany; E-Mail: jana@bioinf.uni-leipzig.de; 2 German Centre for Integrative Biodiversity Research (iDiv) Halle-Jena-Leipzig, Deutscher Platz 5E, 04103 Leipzig, Germany; 3 Max Planck Institute for Mathematics in the Sciences, Inselstraße 22, D-04103 Leipzig, Germany; 4 Fraunhofer Institute for Cell Therapy and Immunology, Perlickstrasse 1, D-04103 Leipzig, Germany; 5 Department of Theoretical Chemistry of the University of Vienna, Währingerstrasse 17, A-1090 Vienna, Austria; 6 Center for RNA in Technology and Health, University of Copenhagen, Grønnegårdsvej 3, Frederiksberg C, Denmark; 7 Santa Fe Institute, 1399 Hyde Park Road, Santa Fe, NM 87501, USA

**Keywords:** microRNA, gene loss, genome duplication, innovation, Dollo parsimony, Metazoa

## Abstract

MicroRNAs are important regulatory small RNAs in many eukaryotes. Due to their small size and simple structure, they are readily innovated *de novo*. Throughout the evolution of animals, the emergence of novel microRNA families traces key morphological innovations. Here, we use a computational approach based on homology search and parsimony-based presence/absence analysis to draw a comprehensive picture of microRNA evolution in 159 animal species. We confirm previous observations regarding bursts of innovations accompanying the three rounds of genome duplications in vertebrate evolution and in the early evolution of placental mammals. With a much better resolution for the invertebrate lineage compared to large-scale studies, we observe additional bursts of innovation, e.g., in Rhabditoidea. More importantly, we see clear evidence that loss of microRNA families is not an uncommon phenomenon. The Enoplea may serve as a second dramatic example beyond the tunicates. The large-scale analysis presented here also highlights several generic technical issues in the analysis of very large gene families that will require further research.

## 1. Introduction

MicroRNAs (miRNAs) are an important class of endogenous, small, non-coding RNAs that has been described in almost all animals and plants, as well as several clades of unicellular eukaryotes. They play a key role in post-transcriptional gene silencing via targeting a substantial fraction of mRNAs [1]. Their function depends on the presence of the evolutionarily even older RNA interference pathways, through which double-stranded RNA can inactivate cognate sequences; see [2,3] for reviews. Canonical miRNAs are produced through a common processing pathway starting from pol-II-transcribed primary precursor transcript, the pri-miRNA. Hairpin-shaped precursors, pre-miRNAs, are extracted in the nucleus. These products are processed further into miRNA/miRNA* duplexes in a manner that differs substantially between plants and animals; see, e.g., [4] for a recent review. In the final step, the single-stranded mature miRNA is incorporated into the Argonaut complex.

miRNA precursors satisfy rather stringent structural constraints that also substantially differ between animals and plants, strongly suggesting that animal and plant miRNAs have been independent evolutionary innovations that make use of the RNAi machinery in functionally analogous ways. In fact, the innovation of miRNA-like endogenous RNAs seems to have occurred multiple times also in diverse unicellular eukaryotes: despite functional analogy, there is no evidence for any homology between the plant miRNAs, animal miRNAs and the miRNAs reported for protozoa, including trypanosomes [5], toxoplasma [6] and slime molds [7]. In the case of *Giardia*, snoRNAs can take on miRNA-like functions [8,9].

Most miRNAs are among the most highly-conserved genetic elements, at least in animals and plants. Not only the short mature sequence, but the entire precursor is usually under strong stabilizing selection [10], so that the evolutionary origin of individual miRNAs can be traced back in time with high accuracy [11]. Like other gene families, miRNAs are also prone to forming paralogs [12,13], with the result that many miRNAs appear as members of families as homologs, which is also the basis of the miRBase nomenclature [14]. A series of investigations into the phylogenetic distribution of miRNA families led to the conclusion that miRNAs are infrequently lost at the family level and, thus, serve as excellent phylogenetic markers [15,16,17,18]. Although, the massive restructuring of the miRNA complement in tunicates has been recognized as an important exception to this rule [19]. A recent rigorous statistical assessment of the phylogenetic utility of microRNAs [20] furthermore reports high levels of homoplastic loss and sampling errors, casting doubt on miRNAs as phylogenetic markers.

The innovation of new miRNA families appears to be an on-going process, leading to a large number of very young and even species-specific miRNAs [21,22]. A detailed study in fruit flies estimated an innovation rate of as many as 12 new miRNA genes per million years [23], in line with earlier hypotheses that hairpins that can be processed as miRNA precursors frequently appear by chance in essentially random RNA sequences [12,24,25]. Only a tiny fraction of the new miRNAs, however, are ever placed under stabilizing selection, and even fewer are retained in the long run. As a general trend, evolutionarily young miRNAs tend to have low expression levels and evolve faster than their older and more highly expressed counterparts [26,27]. Apparently, evolution is slowed down later on by increasing the selection pressure through the gradual acquisition of more target sites, which, at some point, becomes protective against miRNA loss [28]. The net gain of such permanently-retained miRNA families is only one per several million years, consistent with the comparison of the miRNA complements between metazoan phyla. The general trend of expanding the miRNA repertoire in most lineages appears to correlate with increasing morphological complexity [11,15,16,28,29,30,31], while massive morphological simplification, as in the case of tunicates, seems to be accompanied by the loss of miRNA families [19,32].

Large-scale comparative analyses of animal miRNA evolution have revealed several bursts of miRNA evolution, most notably one associated with the origin of the vertebrates and another one at the root of the placental mammals [11,16,33]. Here, we reevaluate the history of animal miRNAs in light of the recent massive increase in available data. On the one hand, a wide variety of animal species have been surveyed for miRNAs using RNA-seq, while the number of sequenced animal genomes also has more than tripled compared to earlier work. Hence, we now have a database that is much less biased and allows more fine-grained phylogenetic resolution in tracing the origins of an miRNA family. This also serves as a starting point for quantifying the losses of miRNA families.

## 2. Materials and Methods

### 2.1. MicroRNA Detection

The starting points are all metazoan miRNA families stored in miRBase 21 [34]. This database holds 21,263 miRNA precursor sequences for 115 animals species. While 14,712 are organized into families, the remaining 6551 pre-miRNAs are marked as species specific. This leads to a total number of 1415 miRNA families according to miRBase. For 18 of the 115 species, no genome sequence is publicly available, so the presence or absence of miRNA families beyond those reported in miRBase cannot be tested. These species were removed from the final analysis, even though their known miRNAs were used as seed sequences for homology search. We additionally downloaded 44 animal genomes from public sources, like NCBI and ENSEMBL, for which, so far, no miRNAs had been published, resulting in 159 metazoan genomes as targets for homology search. A detailed list can be found in the Supplemental Material together with the numbers of known miRNAs.

In the first pass, homologous precursor sequences of all miRNAs were searched via NCBI blast [35] in *all* 159 genomes. The threshold for blast searches was set to *E* *≤* 10*^−^*^10^, and default values were used for all other parameters. Each blast hit must contain the mature miRNA sequences, and its overall length must at least cover 90% of the length of the query. Candidate sequences then were extracted and aligned with their queries with mlocarna [36] to ensure an optimal (simultaneous) alignment of the sequence and secondary structure. Forty one annotated pre-miRNA sequences were not recovered in any of the available genome sequence. Thus, these miRNA families were excluded from further analysis. In addition, we also excluded 46 miRNA families that have more than 100 copies per species. This leaves a total of 1328 miRNA families with member sequences distributed across the phylogenetic range of 159 animal species.

### 2.2. MicroRNA Age

**Figure 1:**
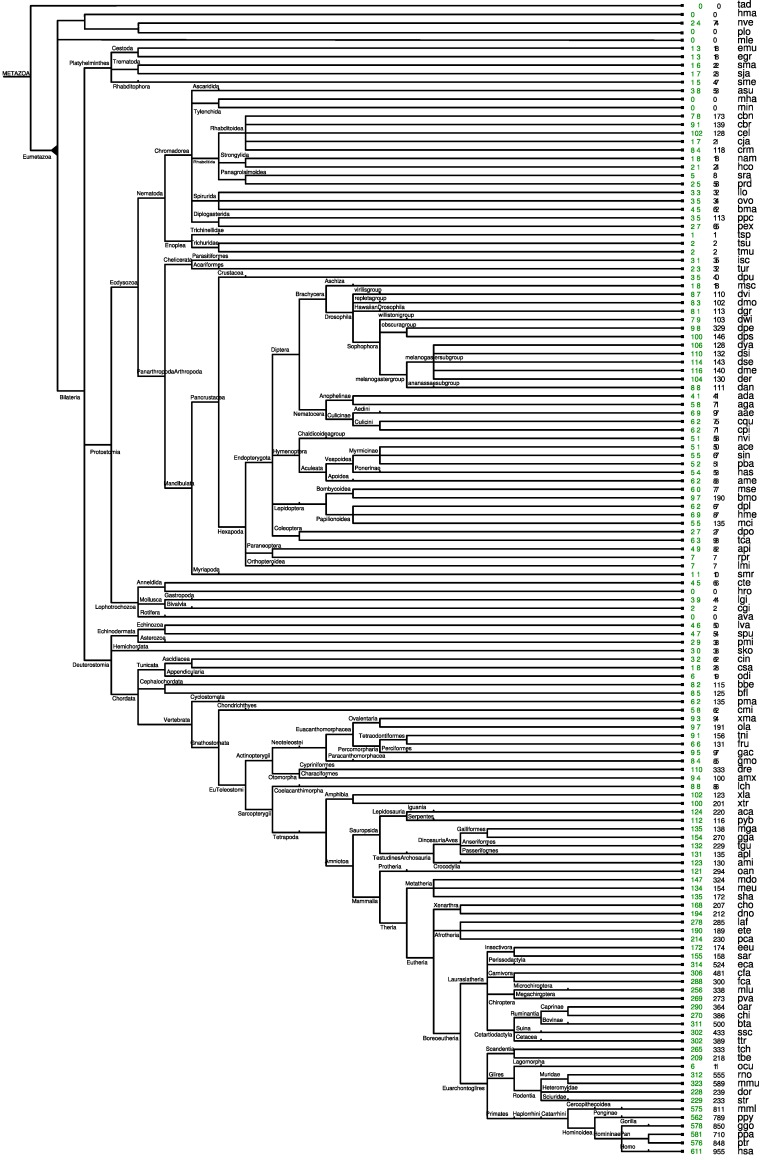
The phylogenetic tree constructed from recent literature on metazoan phylogenetics. The species are assigned to the leaves; see Supplementary 1 for the mapping of the abbreviation, full name and used genome release. Green numbers at leaf nodes refer to the number of miRNA families; black numbers count the miRNA genes within these families (including both known and additional homologs).

The age of an miRNA family is dated relative to the phylogenetic tree *T* shown in Figure 1. It has been produced as a “near consensus” of the recent literature on metazoan phylogenetics; see, e.g., the recent book [37] on the topic. In particular, we adopt Olfactores, as well as Ecdysozoa and Lophotrochozoa and monophyletic groups. The origin of a particular miRNA family *m* is estimated as the branch leading to the last common ancestor (LCA) of all observed family members. We denote the corresponding subtree of *T* by *T_m_*. By abuse of notation, we denote the first node following the origin also by *m*, *i.e.*, *T_m_* is the subtree rooted in *m*.

Since our data curation is rather stringent, it is unlikely that we include false-positive family members. The LCA of an miRNA family is therefore a conservative estimate for the evolutionary origin. Although miRNAs that are similar in sequence may originate multiple times, this effect is mostly observed in repeat-derived families [38,39], which we excluded from our analysis. Thus, we can employ a form of Dollo parsimony, *i.e.*, each miRNA family appears exactly once, but may be deleted multiple times in subtrees. Following the parsimony principle, we assume that an miRNA family is lost in each branch, leading to a maximal subtree (below the family’s origin) in which no family member is observed. Due to our conservative data curation and the incompleteness of some genome assemblies, our estimates for losses will unavoidably be confounded by the bias towards false negatives. Due to the extreme conservation of miRNAs, however, false negatives arising from sequence divergence are extremely unlikely among closely-related species. Incomplete genome assemblies are thus a more plausible source of false negatives.

To efficiently compute the LCA of miRNA families, we first determine a preorder for (an arbitrary ordered representation of) *T* . For each family *m*, we then search (in linear time) for the first leaf *p* and the last leaf *q* (according to this preorder) in which the miRNA family is present. The LCA is then the first node at which the path from *p* and *q* to the root coincide. Thus, the LCA is determined in linear time for each family.

### 2.3. Gains and Losses of Paralogs

We are interested not only in the age and in the losses of an entire miRNA family, but also in the turnover of paralogs. To this end, we need to compute, for each miRNA family *m* and each interior node *v* of the subtree *T_m_* of *T* below the origin of the miRNA family *m*, the number of paralogs. Denote by *u* ⊳ *v* that *u* is a child of *v* in *T*. Furthermore, let *S_kv_* denote the parsimony score at node *v* subject to the constraint that *k* paralogs of miRNA *m* were present at *v* and *v* ∈ *T_m_*. Since the miRNA family does not exist outside *T_m_*, all subsequent computations can be restricted to *T_m_*. This problem can be solved by dynamic programming [40].

Here, we employ a version of Sankoff’s parsimony algorithm, which makes it easy to obtain the most parsimonious estimates for the interior nodes [41]. On the leaves, the parsimony scores are initialized by the data: *S_k__ℓ_* = 0 if *k* is the measured number of paralogs in leaf *ℓ* of *T*, and *S_k__ℓ_* = ∞ otherwise. Furthermore, let *P_v_* denote the maximum number of paralogs in the subtree *T_v_* with root *v*. Of course, *P_ℓ_* equals the measured number of family members for each leaf *ℓ*. For the interior nodes, we have the recursions:

(1) $$P_{v}=\underset{u⊳v}{\text{max }}P_{u}$$

Now, we are in the position to compute the general values of *S_kv_*. To enforce the Dollo constraint, we use:

(2) $$S_{0v}=\left\{ \begin{array}{l} 0\;\;\;\;\;\text{if }P_{v}=0\\ \infty \;\;\;\text{if }P_{v}\geq 1 \end{array}\right.$$

so that a node *v* ∈ *T_m_* cannot be assigned 0 family members when a family member has been recorded in the subtree *T_v_* below *m*. Note that *P_m_* denotes the maximum number of paralogs.

For *k* *≥* 1, we have the general recursion:

(3) $$S_{kv}\;=\sum_{u⊳v}\overset{P_{m}}{\underset{j=0}{\text{min }}}(S_{ju}\;+\;\delta_{u}(j,k))\;.$$

Here, *δ_u_*(*j,k*) denotes the score of changing the number of miRNAs from *k* to *j* between the father *v* of *u* and the child *u*. In the simplest case, *δ_u_*(*j,k*) = *|k* *−* *j|* is just the number of miRNAs inserted by duplication or deleted. In more general models, duplications (*j* *−* *k*) *>* 0 and losses (*j* *−* *k*) *<* 0 might be treated differently and with a node-dependent weight. Here, we use the simple counting score. It is worth noting that in deriving recursion Equation (3), we have made use of the fact that, in general, min*_k__′__,k__″_*(*a_k__′_*+ *b_k__″_*) = min*_k_a_k_*+ min*_k_b_k_*, so that score increments for the children of *v* can be minimized independently. It is easy to see, furthermore, that the most parsimonious solution cannot have more miRNAs at interior nodes than observed at any of the leaves, *i.e.*, we can restrict *k* *≤* *P_m_*.

The score of the most parsimonious scenario is *S* = min*_k_S_km_*. The most parsimonious solution for the number of paralogs at each interior node *v* is then obtained by backtracing. The edges at which miRNA families go extinct are those for which the parent has *k >* 0 and the child has *k* = 0 in the most parsimonious solution.

### 2.4. ePoPE: Efficient Prediction of Paralog Evolution

Our newly developed software, ePoPE, provides a C implementation of the algorithm outlined in Section 2.3. It is designed specifically for the analysis of gene family data, which are usually available in the form of sequence alignments obtained from homology search. Thus, ePoPE takes as input a phylogenetic tree and a sequence alignment that use consistent taxon labels. The software provides two modes of operation: In addition to the parsimony mode, which computes the most parsimonious assignment of counts for all interior vertices of the input tree, a summarizing mode annotates the tree with gain and loss information compiled from the output of the parsimony mode. Annotated trees are provided both as text files and as a postscript interpretation. The auxiliary tool ePoPE-summarize aggregates the text output of ePoPE parsimony computations for different alignments. The ePoPE package is available under the GNU General Public License and can be downloaded at [42].

## 3. Results and Discussion

**Figure 2:**
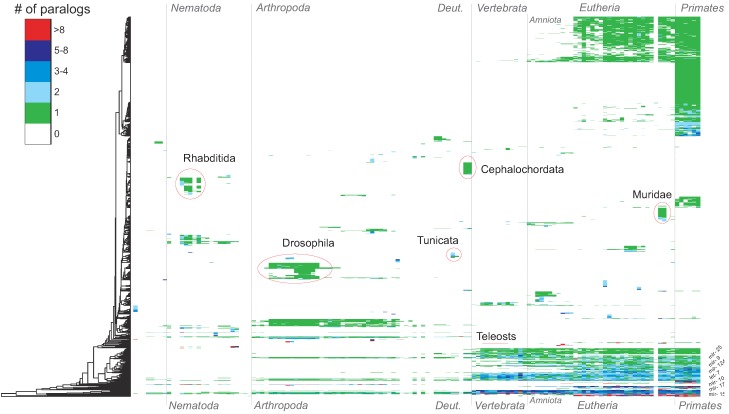
Map of all miRNA families (y-axis) in all analyzed animal species (x-axis). Each cell (*i, j*) represents the number of paralogs within miRNA family *i* in species *j*. The colors indicate this number. The rows have been clustered by co-occurrence (dendrogram at the left side). Beyond several blocks of lineage-specific miRNA families, e.g., in Rhabditida, Muridae or Cephalochordata, the below described bursts of miRNA innovations are also visible here, e.g., at the origin of vertebrates and Eutheria. The miRNA families in the bottom rows cover nearly the complete range of animal species. Indeed, these families comprise many of the evolutionarily old miRNAs, like mir-10, mir-9 and let-7. Furthermore, few miRNA families have more than eight paralogs.

The blast search starting from 19,954 pre-miRNA sequences in the set of animal species resulted in a large number of additional homologs not previously documented in miRBase; see Figure 2. We detected 9482 novel miRNA homologs. Of these, 4363 (*∼*46%) were found in species that were already represented in miRBase. In the additional set of 45 species, we found further 5119 novel homologs. For 452 miRNA families, no additional homologs were found; these seem to be lineage-/species-specific miRNAs. In line with previous studies, we find that miRNAs are continuously integrated into animals’ genomes. There seem to be two dominating types of processes: (i) the *de novo* emergence of new miRNAs from transcribed sequences leads to new miRNA families; and (ii) gene duplications expand the portfolio of paralogs in a given miRNA family. The two mechanisms are thus readily disentangled by the family-wise census of miRNA families reported here.

**Figure 3:**
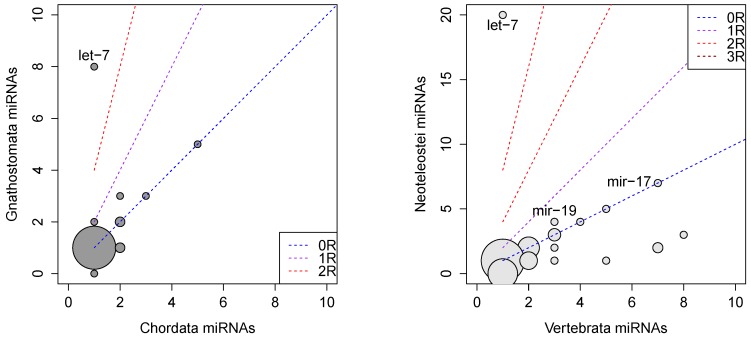
Estimated number of paralogs of miRNA families in the chordate ancestor and after the vertebrate-specific 1R/2R (l.h.s.) and the teleost-specific 3R (r.h.s.) genome duplications. The size of the circles is proportional to the number of miRNA families with given numbers of paralogs in the ancestral state and the node following the gene duplication. The lines have slopes of 1, 2 and 4, respectively.

The software ePoPE introduced here implements a variant of Sankoff’s parsimony algorithm using the Dollo variant that excludes the loss and re-gain of an miRNA family along the same lineage. It was designed specifically for studying the evolution of gene families with variable numbers of paralogs, for example miRNAs. With its help, we identify the last common ancestor of each individual miRNA family and find the most parsimonious estimate for the number of paralogs. As expected, we observe a significant increase in the number of miRNA paralogs at the branch leading to the ancestral gnathostome, vertebrate and teleost; see Figure 3.

The 1R/2R pair of genome duplications has typically not increased the number of paralogs in ancestrally-present miRNA families by the expected factor of four. In fact, a large fraction still shows only a single paralog. This suggests that most of the duplication-related additional copies have been lost again quickly after the duplication event. A similar pattern is observed for the teleost-specific 3R duplication, albeit the resolution is poor, since no actinopterygian lineage that does not share the 3R duplication is included in the present dataset.

The branches harboring the genome duplications are also associated with massive gains of novel miRNA families. The bursts of innovation at the root of the vertebrate tree were already observed previously based on much less extensive datasets [11,16]. The first two rounds of genome duplications are associated with the increase in morphological complexity during early vertebrate evolution; see [43] and the references therein. These duplication events in and of themselves of course do not explain the concurrent bursts in novel miRNA families. The increased plasticity of the genetic system caused by the increase in redundancy, however, makes it generically favorable to integrate additional regulators. This process favors RNA-based regulators [44] and, hence, also the inclusion of novel miRNAs. Interestingly, many of the vertebrate or gnathostome-specific miRNA families appear with multiple paralogs, suggesting that their origin precedes the duplication and that the retention of their paralogs was favored by temporarily-reduced selection pressures. Again, we observe a similar pattern for the branch containing the teleost-specific 3R duplication.

**Figure 4:**
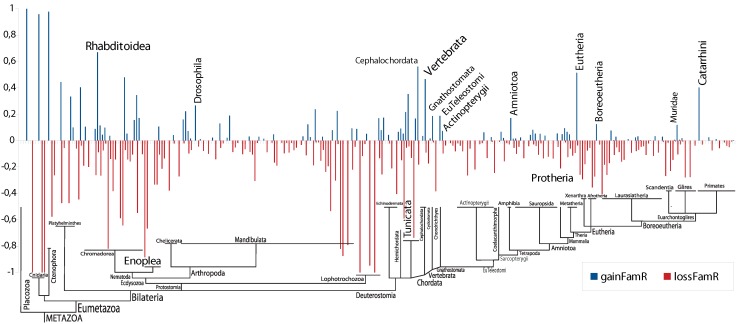
Relative number of gains and losses of entire miRNA families during metazoan evolution. The relative gain is the number of gained miRNA families compared to the observed number of miRNA families. The relative loss describes the number of lost miRNA families compared to the number of miRNA families in the parent node of the phylogenetic tree.

In protostomes, we observe further bursts of innovation of novel miRNA families at the ancestor of “free-living” nematodes, Rhabditoidea and at the split of drosophilids. Beyond the massive gains of miRNA families at the ancestral gnathostome, vertebrate and teleost in deuterostomes, additional peaks in miRNA family innovation can be assigned to the ancestral lines of: (1) Amniota, the egg laying animals; (2) Eutheria, the placental mammals; (3) Boreotheria, the group comprising the Superprimates and the Laurasiatheria; (4) Muridae, the group containing mouse, rat and gerbil; and (5) Catarrhini, the Old World Monkeys, including apes and humans; see Figure 4. All of these branches are associated with major increases in morphological complexity in these lineages of animal species [45].

Although miRNA families have been advertised as particularly homoplasy-poor phylogenetic characters [15], we observe appreciable levels of loss, not only of individual paralogs, but of entire miRNA families. This is in agreement with a recent report on high levels of gene loss and missing data that make the use of miRNAs for phylogenetic purposes much more problematic than previously thought [20]. The large number of additional family members found by homology search in our study shows that missing data are a major contribution to the uncertainties uncovered in [20]. Despite the extensive homology search performed in our study, massive lineage-specific losses remain. Although we cannot rule out that highly-derived homologs have escaped our search, it appears unlikely that the apparent losses are completely accounted for by missing data.

Gene loss is enhanced after large-scale duplications [46,47]. In part, this is explained by the approximate redundancy of recent duplicates; in part, it appears to be a consequence of ongoing changes that have been ongoing long beyond the duplication event itself. A well-understood example is the differential loss of HOX genes throughout vertebrate evolution [48]. We do not observe concentrations of family loss in the aftermath of the genome duplications. Instead, we observe an approximately constant loss rate across nearly the entire metazoan tree; see Figure 4.

A notable exception is the subphylum, Tunicata. These closest relatives of the vertebrates have compact, rapidly-evolving genomes [49] and have been reported to have undergone a drastic reorganization of their miRNA system [19]. miRBase reports 441 pre-miRNAs for this clade (*Ciona intestinalis*, 348; *C. savignyi*, 27; *Oikopleura dioica*, 66), of which only 105 (24%) are organized into 36 families. Our blast search yielded only three additional orthologs in *C. savignyi* within the three tunicates, suggesting a highly species-specific set of independently-innovated miRNA families. The gain/loss analysis with the ePoPE program confirmed the expected loss of a high number of miRNA families in the ancestral urochordate, followed by the innovation of a large number of novel miRNA families that show no homology with miRNAs outside of tunicates.

The single miRNA family that is conserved throughout Eumetazoa is *mir-10* (which includes mir-10, as well as its distant paralogs, mir-100, mir-99, mir-51 and mir-57 in the current miRBase nomenclature). It also comprises the most ancient miRNA system in animals. Within Protostomia, miRBase lists mir-10 and mir-100 orthologs for Lophotrochozoa and arthropods, while mir-51 and mir-57 families are annotated in nematodes (with the exception of *Brugia malayi* mir-100 miRNA). The precursor sequences have diverged substantially, while the mature sequences appear to be conserved, sharing at least the seed sequence. The nematodes comprise two major groups, the Enoplea and the Chromadorea [50]. Only the latter has been the target of experimental surveys for miRNAs. Computationally, we recover mir-100 orthologs in the three Enoplea species, *T. spiralis*, *T. suis* and *T. muris*, and in the subgroup Spirurida of Chromadorea, with *Loa loa* and *Onchocerca volvulus* (containing *Brugia malayi*).

The only other miRNA family that we detect in Enoplea is mir-9, which also belongs to the evolutionarily old miRNA families that can be traced back to the “Urbilateria”. Twenty-one additional miRNA families have originated before the divergence of Enoplea and Chromadorea and are attested in the latter group. None of them could be detected in the Enoplea, suggesting a massive loss of miRNAs in the ancestral lineage of the Enoplea (see Figure 4), which is as dramatic as the losses reported for tunicates [19]. It remains a task for future experiments to see if the losses have been compensated by equally extensive innovations of novel miRNA families.

In Lophotrochozoa, it is hard to decide whether we see a large number of individual *de novo* innovations of miRNA families for *L. gigantea* and *C. capitata* or a large number of lost miRNA families in the remaining lophotrochozoan species. The group of K. Peterson annotated several of these miRNAs in *L. gigantea* and *C. capitata* [18,51]. In addition to these two organisms, we searched the genomes of the annelid *Helobdella robusta*, the mollusc *Crassostrea gigas* and the rotiferan *Adineta vaga*. With the exception of mir-9, which we computationally detected in *C. gigas*, neither the (other) miRNAs that are reported for *L. gigantea* and *C. capitata* nor those that have their LCA before the split of Lophotrochozoa are found in these species. Missing data may be an issue here, because these genomes have just become publicly available in their first (draft) assembly; they all have been reported to be repeat-rich and, thus, hard to assemble [52,53,54].

The quantitative approach pursued here is sensitive to several sources of error. Incomplete genome sequences may also account for some apparent species-specific losses. Even with perfect data, however, there are inherent limits to the sensitivity of homology search for non-coding RNAs [55]. Both the relatively fast turnover of nucleic acid sequences (at least outside the mature miR sequence) and the very short size of the pre-miRNAs can be limiting. This may lead not only to false negatives, but also false-positive innovation events through the erroneous assignment of different miRNA family names to distance homologs. A case in point is *O. dioica* *odi-mir-1473*, which was identified as a *mir-100* homolog in [13] by manual curation. We have opted here for a Dollo parsimony approach, since we may assume that the same miRNA sequence will not appear twice by chance. There are, however, potential mechanisms that will create very similar miRNAs by exaptation from repetitive elements [38] or possibly abundant pseudogene families [56]. We have here attempted to reduce the impact of such cases as much as possible be removing all miRNA families with large copy numbers within a single genome.

## 4. Conclusions

We have attempted here to provide an updated account of miRNA evolution in animals using an essentially automatic work flow and a quantitative evaluation of gain and loss. The feasibility of this approach is based on two strong assumptions: (1) the correct phylogeny of animals is known at least w.r.t. all species with completely sequenced genomes; and (2) reliable and complete presence/absence data for miRNAs are obtainable for all species under consideration. The restriction of the metazoan tree to the fully sequenced genomes is, indeed, fairly well known, and for most nodes, a consensus opinion can be adopted [37]. We resort to considering some of the contested nodes as multifurcations.

The second assumption, however, is violated at several levels. First, miRNA sequencing data are available only for a rather limited subset of the species. Innovations of novel miRNA sequences cannot be estimated along these lineages. On the other hand, estimates of losses are affected by the limitations of homology search. While we can be confident that false-positive homologs are rare due to stringent filtering criteria, we are likely to miss some distant, highly-diverged homologs. The often relatively poor status of sequence assemblies is a likely source of additional false positives. Despite all of the limitations of the homology search strategy employed here, we emphasize that this is a necessary step, and a quantitative survey cannot be built on current annotation alone. As a case in point, RefSeq coverage of protein-coding genes differs by up to an order of magnitude between very closely-related mammalian genomes [57] depending on the maturity of the annotation efforts.

By using a parsimony approach to infer the number of paralogs in ancestral nodes, we make a third assumption: (3) losses and duplications of members of the same miRNA family within the same internal edge of the phylogenetic tree do not occur. It is clear that this property is sometimes violated. A more accurate analysis, however, would require a high-quality orthology annotation for miRNA paralogs. We suspect that this could be achieved at least in part by taking syntenic conservation into account. For several well-studied larger miRNA families, however, extensive manual curation has turned out to be required for this purpose [12,13,58]. We have therefore shied away from this level of resolution.

Despite all of these caveats, we can draw several conclusions from our analysis: (1) innovation is an ongoing process in all animal clades investigated so far; (2) despite the ubiquity of the process, there are several bursts of miRNA birth, in most cases associated with major morphological and physiological innovations; in this respect, our data confirm and refine previous analyses; and (3) our data strongly suggest that miRNAs are not as evolutionarily stable as commonly expected; see also [20]. Substantial losses of ancestral miRNA families are, in fact, not uncommon, in particular the evolution of invertebrates. Even accounting for incomplete data and possible divergent sequences, there are clear indications of a major loss in Enoplea, where only two evolutionarily old miRNAs are conserved. Although, there are many miRNAs annotated in lophotrochozoan species, only mir-9 has its last common ancestor outside of this group. In other words, the majority of miRNAs that have originated from the ancestral bilaterian seem to have been lost here.

## Supplementary Files

Supplementary File 1

Supplementary File 2
